# Effects of Negative Meta-Stereotype on the Doctor–Patient Relationship: The Role of Imagined Intergroup Contact

**DOI:** 10.3390/bs16050819

**Published:** 2026-05-19

**Authors:** Yanli Zhu, Mengzhu Jiang, Fan Feng, Jingru Sun

**Affiliations:** 1 Department of Psychology, School of Education, Zhengzhou University, Zhengzhou 450001, China; zhuyl99@zzu.edu.cn (Y.Z.);; 2 Department of Psychology, School of Education, Xuchang University, Xuchang 461000, China

**Keywords:** negative meta-stereotype, intergroup anxiety, doctor–patient trust, doctor–patient relationship, imagined intergroup contact

## Abstract

This study aims to explore the relationship between negative meta-stereotypes, intergroup anxiety, doctor–patient trust, and doctor–patient relationships and investigate the intervention impact of imagined intergroup contact through two experiments. Study 1 examined whether intergroup anxiety and doctor–patient trust sequentially mediate the effect of negative meta-stereotypes on the doctor–patient relationship. Two hundred participants were randomly assigned to a negative meta-stereotype activation or control group, and completed measures of intergroup anxiety, doctor–patient trust, and doctor–patient relationship quality. Study 2 built on these findings by testing imagined intergroup contact as a potential intervention. Following negative meta-stereotype activation, 184 participants were randomly assigned to one of four conditions: no imagination, imagined landscape, imagined contact with a professionalism-focused physician, or imagined contact with a care-focused physician. Study 1 revealed that negative meta-stereotype activation significantly increased intergroup anxiety, decreased doctor–patient trust, and impaired the doctor–patient relationship. Importantly, intergroup anxiety and doctor–patient trust played a chain mediating role in this relationship. Study 2 demonstrated that only imagined contact with a caring physician (but not a purely professional one) significantly reduced intergroup anxiety, enhanced doctor–patient trust, and improved the doctor–patient relationship. These findings provide evidence consistent with a serial emotional-cognitive pathway from intergroup anxiety to doctor–patient trust through which negative meta-stereotypes may impair the doctor–patient relationship, and by pinpointing emotional care as the core component that makes imagined intergroup contact effective in this context.

## 1. Introduction

In the context of social transformation and medical model change, the incidents of doctor–patient conflict in China remain high (Wang & Du, 2023). How to establish a harmonious doctor–patient relationship has become the focus of society. The doctor–patient relationship in China is a crisis-ridden relationship model that combines both interpersonal and intergroup dimensions (Chai, 2017). Meta-stereotypes are activated when group identity becomes salient, and individuals may be evaluated by outgroup members (D. B. Ma & Yao, 2022). A meta-stereotype is a personal belief about the stereotypical impression that out-group members have of the in-group to which they belong, with positive, neutral, and negative valence. It is contextual in nature. Its activation varies according to the out-group and has profound effects on intergroup relations (Gómez & Huici, 2008; Y. Ma et al., 2022). Studies have generally found that negative meta-stereotypes bring about negative effects such as intergroup anxiety and negative intergroup attitudes (Finchilescu, 2010; L. Xu et al., 2018). Most current studies focus on different ethnic and gender groups (W. He et al., 2014), with fewer examining meta-stereotypes between doctors and patients. Some studies have confirmed the negative impact of meta-stereotypes on doctor–patient relationship (W. He et al., 2020), but the pathway of this impact remains unclear. Whether intergroup contact between doctors and patients could alleviate the negative effects of negative meta-stereotypes requires verification.

### 1.1. Negative Meta-Stereotypes and the Doctor–Patient Relationship

Although some studies have shown that the activation of negative meta-stereotypes can produce positive outcomes, such as enhanced prosocial behavior and generosity (Hausmann, 2014; Hopkins et al., 2007; Tung, 2019), the predominant finding indicates that meta-stereotype activation generally maintains valence-consistent effects (Finchilescu, 2010). This means that in most cases, the impacts triggered by meta-stereotypes are relatively stable and consistent in terms of nature (positive or negative) and degree. For example, if the meta-stereotype is negative, it tends to continuously produce negative effects, such as reducing individuals’ self-efficacy and triggering tensions between groups. On the other hand, if the meta-stereotype is positive, it usually brings about positive outcomes, like enhancing individuals’ self-confidence and promoting cooperation among groups. Negative meta-stereotypes may induce adverse consequences (Dong et al., 2022). Individuals holding such stereotypes often avoid intergroup contact due to fear of negative evaluation (Gaertner & Dovidio, 2014; Huang et al., 2019; L. Xu et al., 2021). These avoidance behaviors can trigger negative intergroup emotions and impair outgroup evaluations, fostering blame attribution bias that escalates doctor–patient conflict risks (Liu et al., 2024). Within traditional doctor–patient dynamics where patients exhibit low perceived power (Zhang et al., 2020), their vulnerable communication position makes them particularly susceptible to activating negative meta-stereotypes (Y. Ma et al., 2022). Grounded in theory and empirical evidence, we propose the following hypothesis:

**Hypothesis 1.**  *Negative meta-stereotypes held by patients demonstrate significant adverse impacts on the doctor–patient relationship.*

### 1.2. The Mediating Role of Intergroup Anxiety and Doctor–Patient Trust

Intergroup anxiety manifests as individuals’ negative emotional responses during intergroup contact (Stephan, 2014). Finchilescu (Finchilescu, 2010) incorporated meta-stereotypes into the intergroup anxiety framework, positing that negative meta-stereotype activation exacerbates such anxiety. Empirical studies demonstrate that negative meta-stereotypes may (a) elevate physiological arousal, (b) increase intergroup anxiety, (c) promote negative effects, and (d) impair intergroup relations (Davis & Stephan, 2011; Guan, 2017; Kim & Oe, 2009). Crucially, modifying negative meta-stereotypes can mitigate intergroup anxiety (Y. Xu et al., 2024), while heightened anxiety reciprocally reinforces these stereotypes. This bidirectional relationship establishes intergroup anxiety as a pivotal mediator between negative meta-stereotypes and relational outcomes (W. He et al., 2018; Smith et al., 2007; Wohl et al., 2011). Therefore, we hypothesize that:

**Hypothesis 2.**  *Intergroup anxiety may mediate the relationship between negative meta-stereotype activation and the doctor–patient relationship.*

As posited by prior scholarship, “the deterioration of the doctor–patient relationships fundamentally stems from trust deficits” (Sewell, 2015). Doctor–patient trust emerges from both rational appraisal during interactions and social identification processes (Li et al., 2021). For example, when patients form a positive social identification with the professional competence, professional ethics, and other aspects of the doctor group at the social level, during the specific medical treatment process, this identification will be transformed into trust in individual doctors, and they will be willing to believe in the diagnosis and treatment plans provided by doctors. Negative meta-stereotypes activate negative emotions (e.g., anger, anxiety) that drive specific behavioral tendencies such as aggression or social withdrawal (Jiang, 2018). Although empirical evidence remains limited regarding meta-stereotypes’ impact on intergroup trust, extant studies reveal that patients harboring negative meta-stereotypes toward doctors demonstrate heightened skepticism toward medical recommendations and reduced treatment adherence during clinical encounters (Tucker et al., 2015). Thus, we propose the following hypothesis:

**Hypothesis 3.**  *Doctor–patient trust may mediate the relationship between negative meta-stereotype activation and the doctor–patient relationship.*

Critically, theoretical and empirical evidence suggest that intergroup anxiety may serve as a proximal emotional antecedent that subsequently impairs cognitive evaluations of the outgroup, such as trust. The affect-as-information theory elucidates how negative emotions systematically distort social perceptions and thereby undermine trust (Dunn & Schweitzer, 2005; L. Xu et al., 2021). Evidence indicates that negative meta-stereotypes may: (a) induce intergroup anxiety, (b) heighten fairness sensitivity, and (c) amplify negative outgroup expectations (Finchilescu, 2010; Y. Ma et al., 2022). These mechanisms collectively erode interpersonal trust (Bo et al., 2023). The Broaden-and-Build Theory of Positive Emotions posits that positive affective states (e.g., joy, gratitude) broaden individuals’ attentional scope and promote proactive engagement in exploratory behaviors. Conversely, negative emotions (anger, anxiety) signal potential environmental threats, significantly eroding trust levels (Dunn & Schweitzer, 2005; X. L. He et al., 2011). Trust, operationally defined as a psychological state entailing interpersonal risk-taking with positive behavioral expectations toward others (Bo et al., 2023), becomes compromised under heightened intergroup anxiety. Elevated anxiety thus predicts amplified negative outgroup expectations and attenuated trust during intergroup transactions. While prior studies have provided initial evidence that intergroup anxiety and doctor–patient trust each independently mediate the meta-stereotype relationship link (W. He et al., 2020; L. Xu et al., 2021), the sequential process through which these mechanisms operate has not been examined. The present study extends this work by testing a chain mediation model in which anxiety precedes and shapes trust. In summary, the relationship between negative meta-stereotypes and the doctor–patient relationship may be affected first through intergroup anxiety and then via doctor–patient trust. Therefore, we put forward the following hypothesis:

**Hypothesis 4.**  *Negative meta-stereotypes may indirectly affect the doctor–patient relationship through the sequential mediation of intergroup anxiety and doctor–patient trust.*

### 1.3. The Intervention of Imagined Intergroup Contact

Allport’s (1954) seminal Intergroup Contact Hypothesis posits that intergroup interaction—whether direct or indirect—constitutes an efficacious pathway for improving intergroup relations. However, spatiotemporal constraints often render direct contact implementation impractical. Empirical investigations reveal that indirect contact modalities (e.g., knowledge of cross-group friendships or observational learning of positive intergroup interactions) effectively enhance intergroup attitudes and relational dynamics (Vezzali, 2017; Zhou et al., 2019). Crucially, emerging evidence substantiates the beneficial effects of imagined intergroup contact on relational outcomes (Turner et al., 2013), thereby expanding the theoretical framework’s explanatory scope.

Imagined intergroup contact constitutes an empirically validated strategy for improving doctor–patient relationships (Allport, 1954). Grounded in Intergroup Contact Theory, this approach demonstrates three core benefits through active engagement: (a) prejudice reduction, (b) threat mitigation, and (c) trust enhancement (Dunn & Schweitzer, 2005; Schmid et al., 2014). Supporting this framework, Pettigrew and Tropp’s seminal meta-analysis demonstrated contact’s relational benefits (Pettigrew & Tropp, 2006), while Turner et al. (2013) extended these findings to imagined interactions. Defined as the mental simulation of constructive cross-group engagement (Crisp & Turner, 2009), imagined contact exerts three critical effects: (a) reducing intergroup threats, (b) revising negative stereotypes, and (c) updating conceptual frameworks (Guo et al., 2021) (the alteration and optimization of an individual’s cognitive structure and understanding pattern towards other groups), thereby fostering positive relations (Brambilla et al., 2012). Its efficacy spans diverse populations (Gao et al., 2014), evidenced by reduced gender biases and improved attitudes toward sexual minorities (Guo et al., 2021). Crucially, anxiety reduction mediates these attitude changes (Visintin et al., 2024). Despite these advances, an important question remains underexplored: What specific content of the imagined interaction drives these beneficial effects? According to the Stereotype Content Model (Fiske et al., 2002), social perception is organized along two fundamental dimensions: competence and warmth. Most prior studies have employed generic positive interaction scripts, leaving it unclear which element of the imagined contact—perceptions of the outgroup’s professional competence or their interpersonal warmth—is more effective in counteracting the specific emotional and cognitive deficits triggered by negative meta-stereotypes. Social Identity Theory further posits that individuals’ intergroup interaction patterns are shaped by their group membership and perceived outgroup evaluations of their ingroup (Lin et al., 2020). The doctor–patient relationship is essentially an intergroup relationship. The existence of negative meta-stereotypes, such as some negative stereotypical perceptions that patients may hold towards the doctor group, or similar views that doctors may have towards the patient group, is closely related to social identity theory. The activation of negative meta-stereotypes triggers fear of negative appraisal, thereby fostering adversarial orientations toward outgroups. Two specific questions thus warrant investigation. First, can the detrimental effects of negative meta-stereotypes on intergroup anxiety, trust, and relationship quality be experimentally induced in a patient sample? Second, do different types of imagined intergroup contact—specifically, professionalism-focused versus care-focused—differ in their capacity to alleviate these effects? The present research addresses these questions through two experimental studies. Therefore, we propose:

**Hypothesis 5.**  *Imagined intergroup contact can reduce intergroup anxiety triggered by the activation of negative meta-stereotypes, enhance doctor–patient trust, and improve doctor–patient relationships.*

**Hypothesis 6.**  *Intergroup anxiety and doctor–patient trust play a role in the chain mediating effect of imagined intergroup contact on doctor–patient relationships.*

Grounded in theory and empirical evidence, this research investigates dual mechanisms through two experimental studies. Study 1 extends prior research by testing whether intergroup anxiety and doctor–patient trust sequentially mediate the effect of negative meta-stereotypes on the doctor–patient relationship. Experiment 2 verified the role of imagined intergroup contact in alleviating the impact of negative meta-stereotypes by manipulating the types of imagined intergroup contact.

## 2. Experiment 1: The Effect of Negative Meta-Stereotypes on the Doctor–Patient Relationship: The Chain Mediating Role of Intergroup Anxiety and Doctor–Patient Trust

### 2.1. Purpose of the Study 1

Experimental protocols were implemented to (a) examine the impacts of negative meta-stereotype activation on intergroup anxiety, doctor–patient trust, and doctor–patient relationship, while (b) simultaneously assessing the mediating pathways through which intergroup anxiety and doctor–patient trust transmit these effects.

### 2.2. Methods

#### 2.2.1. Participants and Procedure

A one-factor between-participants design was used, calculated using G*Power3.1 for a two-group independent-samples t-test, with a medium effect size (Cohen’s *d* = 0.50, *f* = 0.25) selected at an *α* = 0.05 (two-tailed) significance level. The analysis predicted that 128 participants (numerator df = 1) would be required to achieve 80% statistical power. Through online recruitment, 200 participants voluntarily joined the study, all reporting medical experiences within the past year. These participants were randomly assigned to either a non-negative meta-stereotype activation group or a negative meta-stereotype activation group. Exclusion criteria applied to medical students, healthcare workers, and participants with prior similar experiment experience.

The activation group completed a guided writing task requiring five adjective/sentence descriptions of patient-associated negative meta-stereotypes, while the control group described technological advancements under identical format constraints. The following survey was conducted on “Wenjuanxing”, a professional online survey platform. Ten participants were excluded due to either abbreviated response times or failure to activate negative meta-stereotypes, resulting in final groups of 96 (activation) and 95 (control). Mean ages were 22.77 (*SD* = 2.477) for the activation group and 23.32 (*SD* = 2.744) for controls. Group comparisons revealed no significant differences in age (*p* = 0.150), gender (*p* = 0.653), education level (*p* = 0.056), or health status (*p* = 0.230). Although an effort was made to recruit participants aged 45 and above, preliminary analyses indicated that the experimental effects were not robust in this older subsample, likely due to the small number of eligible participants in that age range. Experiment 2, therefore, focused on a younger, predominantly female sample to ensure adequate statistical power for the imagined contact manipulation.

#### 2.2.2. Measurement Scales

The manipulation of negative meta-stereotypes. Following Owuamalam et al.’s (2013) method, participants in the negative meta-stereotype activation group were instructed, “As a patient, what negative impressions might doctors hold regarding patients’ medical knowledge, personality traits, social status, or lifestyle during clinical encounters? Please describe using at least five adjectives or sentences,” while the control group was instructed, “What are your perspectives on current technological advancements? Provide at least five descriptive adjectives or sentences.”

To assess manipulation efficacy, participants responded to the item: “How do you believe doctors generally perceive patients?” using a 7-point Likert scale (1 = very negative, 7 = very positive), with elevated scores reflecting stronger endorsement of positive meta-stereotypes. Activation success was evaluated through between-group comparisons.

The Intergroup Anxiety Scale. The level of intergroup anxiety was assessed using the 10-item Intergroup Anxiety Scale developed by Stephan (2014). Participants were instructed to indicate the extent to which each emotional state described how they typically felt during interactions with doctors. Example items include “confident” (reverse-scored), “awkward,” and “nervous.” Responses were provided on a 7-point Likert scale ranging from 1 (not at all) to 7 (extremely). Three of the ten items were reverse-scored. After recoding, a total score was computed, with higher scores indicating greater intergroup anxiety. This scale has demonstrated good reliability and validity in both Chinese and international studies. In this study, the Cronbach’s alpha coefficient of this scale was 0.850.

Measurement of doctor–patient trust. The Chinese version of the Wake Forest Physician Trust Scale was used to evaluate the level of doctor–patient trust. Translated into Chinese by Enhong and Yong (2012), this scale consists of 10 items. Participants respond on a 5—point Likert scale (for example, “My doctor will do everything possible to ensure my health”), with 1 representing “strongly disagree” and 5 representing “strongly agree”. The scale includes two dimensions: trust in the doctor’s benevolence and trust in the doctor’s technical competence, and is scored on a 5—point Likert scale. Higher scores indicate a higher level of doctor–patient trust. In this study, the Cronbach’s alpha coefficient of this scale was 0.802.

Measurement of the doctor–patient relationship. The Physician-Patient Relationship Scale (PDRQ-15), developed by Van der Feltz-Cornelis et al. and translated into Chinese by Yang and Wang (2011), was employed to measure the doctor–patient relationship. The PDRQ-15 scale comprises 15 items and 3 dimensions: satisfaction with the doctor, the doctor’s approachability, and attitudes towards medical symptoms. Participants rated each item (such as “My doctor understands me”) on a 5-point Likert scale, with 1 indicating “very strongly disagree” and 5 indicating “very strongly agree”. A higher total score implies a more harmonious doctor–patient relationship. In this study, the Cronbach’s alpha of this scale was 0.856.

#### 2.2.3. Ethical Statement

The study was reviewed and approved by the Ethics Committee of the School of Psychology at Zhengzhou University of China. Written informed consent was obtained from all participants. All procedures were conducted in accordance with the ethical standards of the Human Research Ethics Committee and the Helsinki Declaration.

### 2.3. Results of Study 1

#### 2.3.1. Preliminary Analyses: Descriptive Statistics and Correlation Analysis

A Pearson correlation analysis was conducted to examine the bivariate correlations among the variables under study. According to the results presented in Table 1, perceived meta-stereotypes (higher scores = more positive) were significantly and negatively correlated with intergroup anxiety (*r* = −0.335, *p* < 0.01), while perceived meta-stereotypes were significantly positively correlated with doctor–patient trust (*r* = 0.507, *p* < 0.01) and doctor–patient relationship quality (*r* = 0.497, *p* < 0.01). Moreover, intergroup anxiety was significantly negatively correlated with doctor–patient trust (*r* = −0.507, *p* < 0.01), as well as doctor–patient relationship quality (*r* = −0.468, *p* < 0.01). Finally, Doctor–patient trust was significantly positively correlated with doctor–patient relationship quality (*r* = 0.666, *p* < 0.01).

#### 2.3.2. Negative Meta-Stereotype Manipulation Check in Study 1

The results of the independent samples t-test showed that the activation group’s score on the manipulation test question (*M* = 4.64, *SD* = 0.908) was significantly smaller than the control group’s score (*M* = 5.30, *SD* = 1.217) (*p* < 0.001), indicating that the manipulation was valid.

#### 2.3.3. Between-Group Differences in Intergroup Anxiety, Doctor–Patient Trust, and the Doctor–Patient Relationship

**Figure 1:**
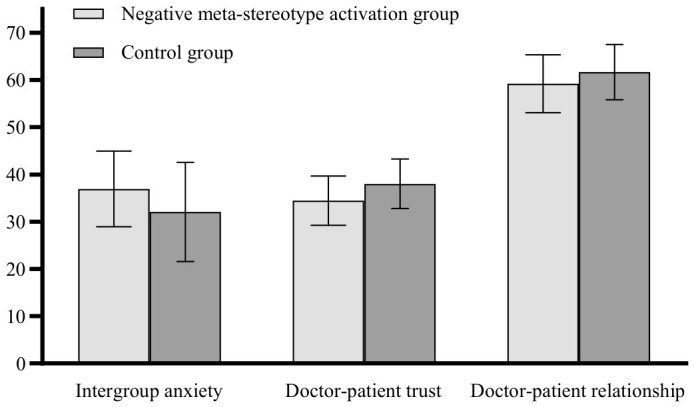
Differences between the two groups of participants on various variables. Error bars represent 95% confidence intervals. Same as below.

Results of between-group ANOVA on intergroup anxiety, doctor–patient trust, and doctor–patient relationship are presented in Table 2. As illustrated in Figure 1, the activation group exhibited significantly elevated intergroup anxiety, alongside trust and impaired relationship quality relative to controls. These findings are consistent with the hypothesis that negative meta-stereotype activation is associated with intergroup anxiety, diminished trust and less favorable relational outcomes in doctor–patient contact.

#### 2.3.4. Mediating Effects of Intergroup Anxiety and Doctor–Patient Trust

The negative meta-stereotype was used as the independent variable, intergroup anxiety, and doctor–patient trust as the mediator variables, and doctor–patient relationship as the dependent variable, and the chain mediation model was established by controlling for gender, education, and age. The results of the regression analysis are presented in Table 3. Negative meta-stereotypes (higher scores = more positive) significantly predicted lower intergroup anxiety (*β* = −0.279, *p* < 0.001) and higher doctor–patient trust (*β* = 0.374, *p* < 0.001). Negative meta-stereotypes also had a significant positive direct effect on the doctor–patient relationship (*β* = 0.199, *p* < 0.01). Intergroup anxiety significantly and negatively predicted doctor–patient trust (*β* = −0.371, *p* < 0.001) and the doctor–patient relationship (*β* = −0.173, *p* < 0.01). Finally, doctor–patient trust was positively associated with the doctor–patient relationship (*β* = 0.475, *p* < 0.001).

**Figure 2:**
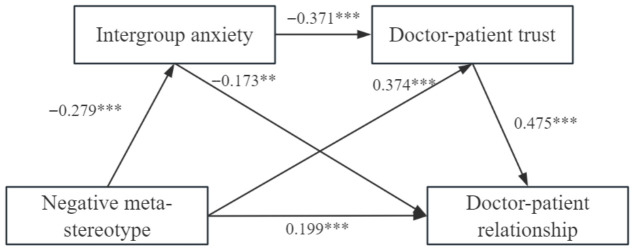
Diagram of the chained intermediary model. ** *p* < 0.01, *** *p* < 0.001.

The study employed the bootstrapping procedure to test the significance of the chain- mediated model. Direct, indirect, and total effects are presented in Table 4 and Figure 2. The indirect effect of negative meta-stereotypes on the doctor–patient relationship via intergroup anxiety was statistically significant (bootstrap estimate = 0.048, 95% CI = [0.009, 0.082]), indicating that intergroup anxiety significantly mediated the relationship between negative meta-stereotypes and doctor–patient relationship quality. Therefore, hypothesis 2 was confirmed. The indirect effect of negative meta-stereotypes on the doctor–patient relationship via doctor–patient trust was statistically significant (bootstrap estimate = 0.178, 95% CI = [0.104, 0.266]), indicating that doctor–patient trust significantly mediated the relationship between negative meta-stereotypes and doctor–patient relationship quality. Hypothesis 3 was confirmed. Moreover, the indirect effect of negative meta-stereotypes on the doctor–patient relationship, via intergroup anxiety and doctor–patient trust (bootstrap estimate = 0.049, 95% CI = [0.022, 0.082]), suggested that intergroup anxiety and doctor–patient trust played a chain mediating role in the relationship between negative meta-stereotypes and the doctor–patient relationship. Therefore, hypothesis 4 was supported, confirming the establishment of chain mediation.

### 2.4. Discussion of Study 1

Study 1 demonstrated that negative meta-stereotypes exert detrimental effects on the doctor–patient relational dynamics. Grounded in social identity theory, individuals derive self-concept from the in-group, fostering preferential in-group evaluations while systematically devaluing out-groups through affective and cognitive biases (Hang, 2016). Crucially, negative meta-stereotype activation compromises self-integrity (Vezzali, 2017), thereby engendering adversarial out-group appraisals and contact avoidance (Lin et al., 2020).

Regression analysis revealed that intergroup anxiety and doctor–patient trust could act as independent mediators and chain mediators between negative meta-stereotypes and doctor–patient relationship, i.e., the activation of negative meta-stereotypes triggered individuals’ anxiety, reduced their trust in doctors, and hindered the development of a harmonious doctor–patient relationship. This result is consistent with the intergroup anxiety model; the affect-as-information model, and the associative semantic network model.

## 3. Experiment 2: The Impact of Imagined Intergroup Contact on the Activation of Negative Meta-Stereotypes

### 3.1. Purpose of the Study 2

Study 1 confirmed that patients’ negative meta-stereotypes significantly increased intergroup anxiety, decreased doctor–patient trust, and affected doctor–patient relationships. Intergroup contact theory suggests that positive intergroup contact can effectively improve intergroup relations. Therefore, Study 2 used an experimental method to manipulate imagined intergroup contact to verify whether imagined intergroup contact could alleviate the high intergroup anxiety, low doctor–patient trust, and deterioration of doctor–patient relations brought about by the activation of negative meta-stereotypes.

### 3.2. Research Methodology

#### 3.2.1. Research Participants

A one-factor between-participants design, calculated using G*Power3.1, was used to select a medium effect (*f* = 0.25) with a significance level of 0.05, predicting that a total of 180 participants would be required to achieve an 80% level of statistical power. Two hundred participants were recruited and randomly assigned to four groups. Exclusion criteria for participants were having similar experimental experience, being medical students or medical workers, ineffective activation of negative stereotypes, and failure of the imagined intergroup contact manipulation. After the test, a total of 16 participants were excluded, leaving 184 participants (112 females). Among them, there were 47 participants in the no-imagination group with a mean age of 26.32 years (*SD* = 8.244), 45 participants in the imagined landscape group with a mean age of 24.87 years (*SD* = 5.102), 47 participants in the imagined professional group with a mean age of 23.87 years (*SD* = 3.201), and 45 participants in the imagined caring group with a mean age of 23.58 years (*SD* = 3.564). There was no significant difference between the four groups of participants in terms of age, health status, and education level.

#### 3.2.2. Measures and Procedures

1. The negative meta-stereotype manipulation, the Intergroup Anxiety Scale, the Doctor–Patient Trust Scale, and the Doctor–Patient Relationship Scale were the same as in Experiment One. Imagined intergroup contact materials refer to Turner et al.’s (2007) imagined intergroup contact paradigm. The detailed instructions for each condition were as follows:
  No-imagination group: No instructions were given; participants proceeded directly to the questionnaires, serving as a comparative baseline.
  Imagined landscape group: Please close your eyes and take 2 min to imagine the following scene: You are sitting on a train, about to travel to a scenic tourist destination. Imagine the scenery you might see along the way. After the imagination exercise, please write down at least three scenes you just imagined.
  Imagined professionalism group: Please close your eyes and take 2 min to imagine the following scene: You are visiting a hospital due to physical discomfort. The doctor treating you is highly skilled and technically proficient. During the consultation, the doctor quickly identifies the cause of your discomfort and formulates an appropriate treatment plan. Throughout the visit, you feel the doctor’s professionalism. After the imagination exercise, please describe in detail which behaviors of the doctor made you feel his/her professionalism (please write at least three points).
  Imagined caring group: Please close your eyes and take 2 min to imagine the following scene: You are visiting a hospital due to physical discomfort. The doctor treating you is warm and approachable. During the consultation, the doctor repeatedly comforts you through words and gestures and formulates an appropriate treatment plan. Throughout the visit, you feel the doctor’s emotional care. After the imagination exercise, please describe in detail which behaviors of the doctor made you feel his/her emotional care (please write at least three points).
2. Experimental procedures. A one-factor between-participants design was adopted, with the independent variable being imagined intergroup contact, the dependent variable being a doctor–patient relationship, and the mediating variables being intergroup anxiety and doctor–patient trust. The procedure of Experiment 2 was as follows: first, negative meta-stereotypes were successfully activated, and to enhance the effect of imagined contact, all groups except the no-imagination group were required to write down the details of their imaginings in as much detail as possible, and then participants were randomly assigned. Among them, the no-imagination group refers to the group in which no imagination activities are conducted, serving as a comparative baseline. The imagined landscape group refers to the group that conducts the imagination of scenery unrelated to the doctor–patient group contact, so as to exclude the influence of general imagination activities. The imagined professionalism group refers to the group that imagines interactions with doctors at the professional level. The imagined care group refers to the group that focuses on imagining the emotional care provided by doctors.

To test the success of the manipulation, participants in the no-imagination group and the imagined landscape group were asked to rate the doctor’s professionalism and emotional concern based on their usual impressions; participants in the imagined professional group and the imagined caring group were asked to rate the doctor’s professionalism and emotional concern in the imagined task after completing the imagined task. Finally, all participants completed the Intergroup Anxiety Scale, the Doctor–Patient Trust Scale, the Doctor–Patient Relationship Scale, and a demographic questionnaire.

### 3.3. Results of Study 2

#### 3.3.1. Descriptive Statistics and Correlation Analysis Between Variables

The results of the mean, standard deviation, and Pearson’s correlation analysis of each variable are shown in Table 5. Imagined intergroup contact was significantly negatively correlated with intergroup anxiety and significantly positively correlated with doctor–patient trust and doctor–patient relationship; intergroup anxiety was significantly negatively correlated with doctor–patient trust and doctor–patient relationship, and doctor–patient trust and doctor–patient relationship were significantly positively correlated.

#### 3.3.2. Negative Meta-Stereotype Manipulation Test Check in Study 2

To test the effect of the manipulation, an independent samples t-test was conducted, and the results showed that there was no significant difference in the degree of negative meta-stereotype activation among the four groups of participants, *F*(3,180) = 0.823, *p* = 0.482 > 0.05.

#### 3.3.3. Imagined Intergroup Contact Manipulation Tests

A one-factor ANOVA was used to test the effect of the imagined intergroup contact manipulation using physician professionalism and caring dimension scores as indicators. The results showed that there was a significant difference between the four groups of participants on professionalism and emotional care, *F*(3,180) = 11.248, *p* < 0.001; *F*(3,180) = 12.242, *p* < 0.001.

**Figure 3:**
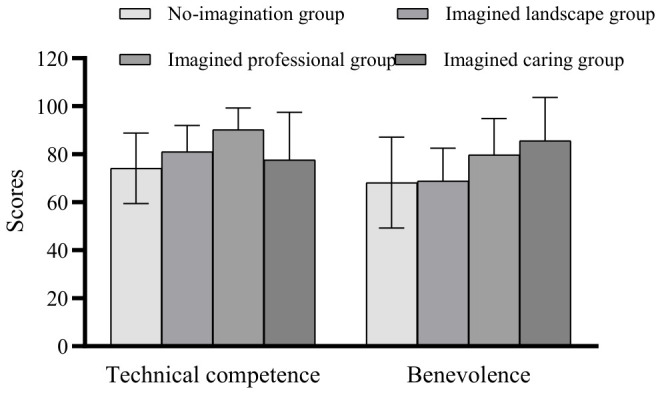
Differences in professionalism and caring scores among the four groups of participants.

In terms of professionalism, the imagined professional group (*M* = 90.28, *SD* = 8.997) was significantly higher than the no-imagination group (*M* = 74.15, *SD* = 14.678), the imagined landscape group (*M* = 81.07, *SD* = 10.870) and the imagined caring group (*M* = 77.69, *SD* = 19.722); in terms of emotional care, the imagined caring group (*M* = 85.69, *SD* = 17.991) was significantly higher than the no-imagination group (*M* = 68.19, *SD* = 18.938) and the imagined landscape group (*M* = 68.19, *SD* = 18.938) and the imagined landscape group (*M* = 68.84, *SD* = 13.688). However, there was no significant difference in caring scores between the imagined caring group and the imagined professional group (*M* = 79.79, *SD* = 15.081), see Figure 3.

One-factor ANOVA was conducted to analyze the intergroup anxiety, doctor–patient trust and doctor–patient relationship scores of the four groups of participants, and the results showed that there was a significant difference between the intergroup anxiety scores of the four groups of participants, *F*(3,180) = 3.199, *p* < 0.05, *ηp*^2^ = 0.051, and there was a significant difference between the doctor–patient trust scores *F*(3,180) = 4.367, *p* < 0.01, *ηp*^2^ = 0.068. There was a significant difference in doctor–patient relationship scores, *F*(3,180) = 3.003, *p* < 0.05, *ηp*^2^ = 0.048, see Table 6.

**Figure 4:**
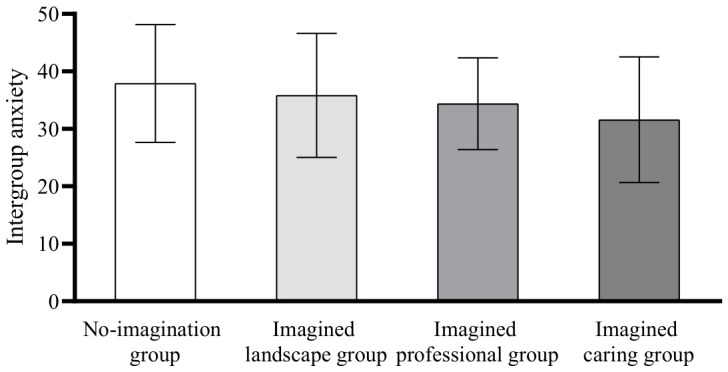
Differences in intergroup anxiety scores.

Post hoc comparisons using the Least Significant Difference (LSD) method showed that there was no significant difference in intergroup anxiety between the no-imagination group and the imagined landscape group (*p* = 0.319); there was no significant difference between the imagined professional group and the no-imagination group (*p* = 0.09), the imagined landscape group (*p* = 0.493), and the imagined caring group (*p* = 0.185); there was a significant difference in the scores of the imagined caring group compared with the scores of the no-imagination group (*p* < 0.01) and the imagined landscape group (*p* < 0.05); the scores of the imagined caring group were significantly lower than the scores of the no-imagination and imagined landscape groups, see Figure 4.

**Figure 5:**
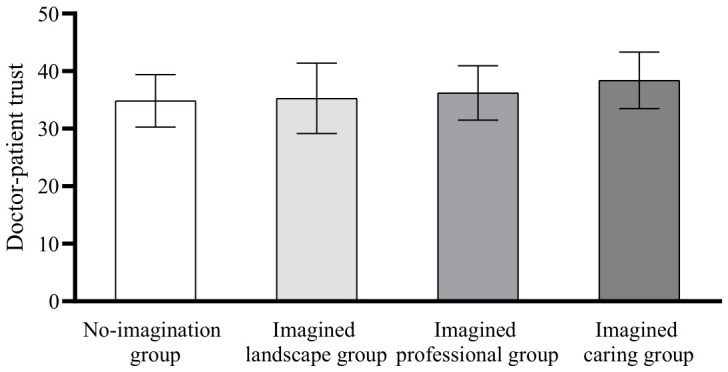
Differences in doctor–patient trust scores.

In terms of doctor–patient trust, there was no significant difference between the no-imagination group and the imagined landscape group (*p* = 0.697); there was no significant difference between the imagined professional group and the no-imagination group (*p* = 0.198) and the imagined landscape group (*p* = 0.376); there was a significant difference between the imagined caring and the no-imagination group (*p* < 0.01), the imagined landscape group (*p* < 0.01) and the imagined professional group (*p* < 0.05), and the imagined caring group doctor–patient trust was significantly higher than that of the no-imagination group, the imagined landscape group and the imagined professional group, see Figure 5.

**Figure 6:**
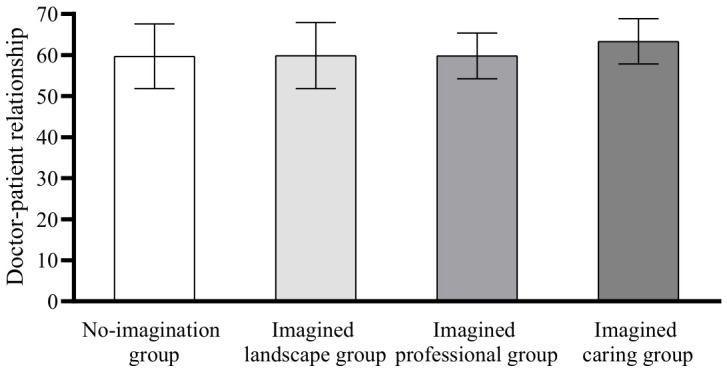
Differences in physician-patient relationship scores.

In terms of the doctor–patient relationship, there was no significant difference between the scores of the no-imagination group and the imagined landscape group (*p* = 0.896); there was no significant difference between the imagined professional group and the no-imagination group (*p* = 0.940) and the imagined landscape group (*p* = 0.955); there was a significant difference between the imagined caring group and the no-imagination group (*p* < 0.05), the imagined landscape group (*p* < 0.05) and the imagined professional group (*p* < 0.05) The doctor–patient relationship score of the imagined care group was significantly higher than that of the no-imagination group, the imagined landscape group, and the imagined professional group, see Figure 6. Therefore, hypothesis 5 was confirmed.

**Figure 7:**
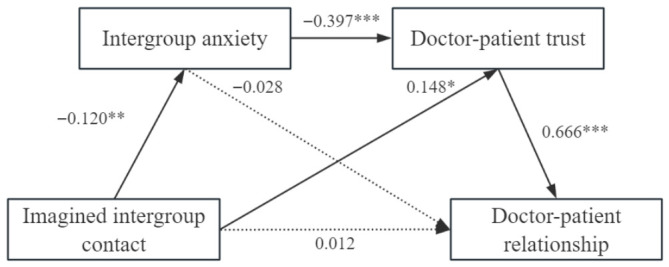
Diagram of the overall mediated effects model. Solid lines indicate significant paths, whereas dashed lines indicate non-significant paths. * *p* < 0.05, ** *p* < 0.01, *** *p* < 0.001.

To further explore the psychological mechanisms by which imagined intergroup contact affects the doctor–patient relationship, the mediating effects of intergroup anxiety and doctor–patient trust were examined with imagined intergroup contact as the independent variable and the doctor–patient relationship as the dependent variable after controlling for gender, education, and age. Given that the independent variable (imagined intergroup contact) was a four-level categorical variable, we adopted a dummy coding approach for the mediation analyses. The no-imagination group served as the reference category, against which the three imagination conditions (landscape, professionalism, and caring) were compared. Three dummy variables were created to represent the contrasts of interest. The omnibus mediation model was chosen over separate pairwise models because it allows for the simultaneous estimation of indirect effects across all contrast groups within a single analytic framework, thereby controlling the familywise error rate and providing a more parsimonious test of the hypothesized mechanisms. According to the mediation effect analysis method proposed by Fang et al. (2017), the overall mediation effect was analyzed first. The results of the overall mediation analysis are shown in Table 7. The independent mediation effect of doctor–patient trust is significant, and the chain mediation effect of intergroup anxiety and doctor–patient trust is significant, as shown in Figure 7, so further relative mediation analysis is needed.

The type of imagined intergroup contact was coded as a dummy variable, and both the mediator and dependent variables were continuous and did not need to be coded. Bootstrap tests were used, and the results of the analysis are shown in Table 8.

**Figure 8:**
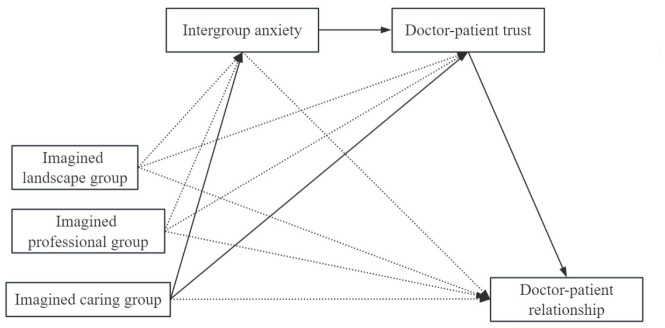
Diagram of the relative mediation effect model. Solid lines indicate significant paths, whereas dashed lines indicate non-significant paths.

Taking the control group (no imagination group) as a reference, the direct and indirect effects of imagined intergroup contact on doctor–patient relationships through intergroup anxiety and doctor–patient trust were not significant in the imagined landscape group and imagined professional group. However, in the imagined care group, the mediation effect value of imagined intergroup contact through intergroup anxiety on doctor–patient relationship was −0.018, with 95% bootstrap confidence interval containing 0, and the mediation effect was not significant; the mediation effect value of imagined care through doctor–patient trust on doctor–patient relationship was 0.309, with 95% bootstrap confidence interval not containing 0, and the mediation effect was significant; and the mediation effect value of imagined care through intergroup anxiety and doctor–patient trust on doctor–patient relationship was 0.309, with 95% bootstrap confidence interval not containing 0, and the mediation effect was significant. The chain mediation effect of intergroup anxiety and doctor–patient trust on the doctor–patient relationship was 0.162, 95% bootstrap confidence interval without 0, and the chain mediation effect was significant; in the imagined care group, the direct effect of imagined intergroup contact on doctor–patient relationship was 0.093, 95% bootstrap confidence interval with 0, and the direct effect was not significant. The above results indicated that in the imagined care group, imagined intergroup contact affected the doctor–patient relationship through the independent mediating effect of doctor–patient trust and the chain mediating effect of intergroup anxiety-doctor–patient trust, as shown in Figure 8. Therefore, hypothesis 6 was confirmed. (All the effect values have been standardized.)

### 3.4. Discussion of Study 2

Experiment 2 provided further evidence for the potential of imagined intergroup contact to counteract the negative effects of negative meta-stereotypes on intergroup anxiety, doctor–patient trust, and doctor–patient relationship by manipulating imagined intergroup contact. After activating the negative meta-stereotype, the intergroup anxiety in the imagined care group was significantly lower than that in the no-imagination group and the imagined landscape group, and the doctor–patient trust and doctor–patient relationship were significantly higher than those in the other groups. This suggests that imagining positive interactions with a doctor who demonstrates emotional care (such as listening attentively to patients’ concerns, showing empathy, and providing emotional support)—a form of imagined contact focusing on interpersonal emotional connection—may reduce patients’ anxiety in intergroup interactions. This, in turn, may alleviate trust barriers caused by negative meta-stereotypes, narrow the psychological distance between doctors and patients at the emotional level, and ultimately contribute to improvements in the quality of doctor–patient relationships. This result is consistent with prior work demonstrating that imagined intergroup contact may play a positive, beneficial role in counteracting the negative impacts brought about by negative meta-stereotypes on intergroup anxiety, doctor–patient trust, and the doctor–patient relationship (Dong et al., 2023). It is noteworthy that the intergroup anxiety of participants in the imagined professional group was not significantly lower than that of the no-imagination group and the imagined landscape group, and the doctor–patient trust and doctor–patient relationship were not significantly higher than that of the no-imagination group and the imagined landscape group, which suggests that merely imagining contact with a technically proficient but emotionally detached doctor (such as one who only demonstrates precise diagnosis or efficient treatment expertise), since it does not address the core mechanisms of emotional trust in doctor–patient relationships, may be insufficient to overcome patients’ psychological defensiveness or negative expectations rooted in negative meta-stereotypes. Thus, it may not effectively counteract the negative impacts on patients’ attitudes and behaviors. The results of the mediating role of intergroup anxiety and doctor–patient trust showed that the direct effect of imagined intergroup contact on the doctor–patient relationship was not significant; however, the relationship could be indirectly associated with imagined contact through the mediating role of doctor–patient trust and the chain mediating role of intergroup anxiety and doctor–patient trust.

## 4. General Discussion

This study discusses the impact of negative meta-stereotypes on the doctor–patient relationship using patients’ perceptions as an entry point, and explores the buffering effect of intergroup contact by incorporating an intergroup perspective. We extend prior work on meta-stereotypes and doctor–patient relationships (W. He et al., 2020; L. Xu et al., 2021) in two main ways. First, rather than examining intergroup anxiety and doctor–patient trust as independent mediators, we tested a serial mediation model in which anxiety precedes and contributes to diminished trust. Second, we deconstructed imagined intergroup contact into two theoretically derived components—professionalism and emotional care—to identify which element is more effective in counteracting the negative effects of negative meta-stereotypes.

### 4.1. The Impact of Negative Meta-Stereotypes on the Doctor–Patient Relationship

The results of Experiment 1 showed that participants in the negative meta-stereotype activation group reported higher intergroup anxiety, lower physician trust, and poorer doctor–patient relationships. Experiment 2 provided further evidence consistent with the negative association between meta-stereotypes and the doctor–patient relationship, which aligns with previous findings (Y. Ma et al., 2022; Kim & Oe, 2009). In the doctor–patient relationship, patients are often in a vulnerable position because vulnerable groups are more concerned about others’ perceptions and often try to guess others’ intentions in their interactions with outgroups, resulting in negative evaluation bias (Gomez, 2002; Wu et al., 2018). In the context of the continuous transformation and new breakthroughs in the global healthcare system, enabling patients to play a more active role in the doctor–patient relationship and establishing a partnership with doctors that can jointly create value may bring about new changes to negative meta-stereotypes and the doctor–patient relationship (Bravo et al., 2015).

### 4.2. The Mediating Effects of Intergroup Anxiety and Doctor–Patient Trust

The results of the mediation analysis in Experiment 1 demonstrated the independent mediating role and chain mediating role of intergroup anxiety and doctor–patient trust between negative meta-stereotypes and the doctor–patient relationship. While prior research has shown that these two factors each independently mediate the meta-stereotype–relationship link (W. He et al., 2020; L. Xu et al., 2021), the present findings extend this work by establishing a sequential pathway in which heightened intergroup anxiety predicts diminished doctor–patient trust. Doctor–patient trust is influenced by patients’ medical experiences (Tucker et al., 2015), and negative stereotype activation may heighten the accessibility of negative expectations associated with those experiences, further impairing trust. The results of the mediation analysis in Experiment 2 showed that imagined emotional caring contact from physicians could influence the doctor–patient relationship through the chain mediation of doctor–patient trust, intergroup anxiety, and doctor–patient trust. There is evidence that both informational and emotional support from physicians can significantly improve the doctor–patient relationship (Wehbe-Janek et al., 2015). In this study, we used the imagined contact paradigm to test the effects of a doctor’s expertise and emotional care on the doctor–patient relationship, and the results showed that compared to imagined contact with a professional doctor, imagined contact with an emotionally caring doctor was associated with lower intergroup anxiety, higher doctor–patient trust, and more favorable doctor–patient relationship ratings. This may be because the participants lacked professional medical knowledge and were unable to judge whether the doctor was reliable or not by the treatment effect during the short contact with the doctor, so they were more inclined to assess the doctor’s ability from the doctor’s attitude, demeanor, and communication skills, etc.

### 4.3. The Role of Intergroup Imagined Contact

Based on Experiment 1, Experiment 2 provided evidence for the intervention effect on the negative impact of negative meta-stereotypes by manipulating imagined intergroup contact. The results of the mediation test showed that among the interventions of intergroup imagined contact on the negative impact of negative meta-stereotypes, the direct effect of intergroup imagined contact on the doctor–patient relationship was not significant. This may be because the doctor–patient relationship is not only affected by negative meta-stereotypes, but also by a variety of factors such as doctor–patient communication and doctor–patient interaction. Although imagined intergroup contact could not directly predict the doctor–patient relationship, it could affect the doctor–patient relationship through the independent mediation of the doctor–patient trust and the chain mediation of intergroup anxiety and doctor–patient trust. This is quite similar to the previous research that found that imagined contact can help repair negative relationships through expectations (Fowler & Gasiorek, 2024). It is noteworthy that the present study found that in the special intergroup relationship of the doctor–patient relationship, doctors’ emotional care could influence patients’ attitudes more than doctors’ professionalism, and this result suggests that doctors’ emotional support may serve as an entry point for improving the doctor–patient relationship. It suggests that for improving intergroup relations that have been damaged by negative emotional and trust-based barriers, the content of the imagined contact is crucial. Generic or purely competence-based positive interactions may be insufficient. Instead, the intervention may need to target the core psychological deficit—in this case, the need for emotional reassurance and a sense of caring.

### 4.4. Shortcomings and Prospects

The results of this study provide some theoretical support for improving the doctor–patient relationship, but this study still has the following shortcomings. Firstly, the variables in this study were manipulated by using guidelines. Although the results of the manipulation test showed that the experimental manipulation was effective, there are still some limitations. For example, patients’ past medical experiences, social opinion environment, and the details of doctor–patient communication will all affect their stereotypical impression of doctors. Future research can adopt a variety of experimental paradigms to allow participants to feel and experience more realistically and observe their reactions; or conduct longitudinal studies to track the psychological changes and behavioral performance of patients in the actual process of medical treatment over a long period of time, so as to more comprehensively explore the relationship between the variables, and to make the results of the study closer to reality and have more application value. Second, the participants in this study focused on young women, but the doctor–patient relationship is affected by various factors such as educational experience, past medical experience, social class, etc. Therefore, a richer sample should be selected in the future to increase the representativeness and generalizability of the results. Thirdly, the current study has a methodological limitation concerning the test of the serial mediation model. As intergroup anxiety and doctor–patient trust were measured simultaneously, the causal ordering of these mediators—where anxiety theoretically precedes and shapes trust—could not be definitively established. While our proposed anxiety → trust pathway is grounded in robust theoretical frameworks, such as the affect-as-information theory which posits that emotional states (anxiety) serve as a heuristic cue that colors subsequent cognitive judgments (trust), we cannot fully rule out alternative directional models with the present cross-sectional data for the mediators. Future research should employ longitudinal designs with multiple waves of data collection, or experimental-causal-chain designs, to verify the temporal precedence of anxiety over trust. Fourth, the manipulation of negative meta-stereotypes relied on a guided writing task with a single-item manipulation check. While this approach has been used in prior meta-stereotype research (Owuamalam et al., 2013) and the manipulation check confirmed a significant between-group difference, a single item may not fully capture the complexity of the meta-stereotype activation process. Future studies could strengthen the manipulation by incorporating multiple manipulation check items assessing different facets of meta-stereotype activation (e.g., awareness, emotional salience, perceived negativity) and by including a pilot validation study to establish the manipulation’s reliability and content validity. Fifth, the control condition in Experiment 1 asked participants to describe technological advancements, a topic that differs from the meta-stereotype activation task not only in stereotype content but also in emotionality, self-relevance, and social salience. The meta-stereotype writing task required participants to reflect on how they believe doctors perceive them as patients, which is inherently self-relevant, socially evaluative, and potentially emotionally evocative. In contrast, describing technological advancements is a neutral, non-self-relevant topic. Thus, the observed between-group differences may partly reflect the general effects of engaging in an emotionally salient, self-relevant task rather than the specific activation of negative meta-stereotypes. While this guided writing paradigm has been used in prior meta-stereotype research (Owuamalam et al., 2013), future studies should employ control tasks that are matched on emotionality and self-relevance or include multiple control conditions to isolate the specific effect of meta-stereotype activation. Sixth, in both experiments, participants who failed the manipulation check were excluded from the analyses. While this is a common practice to ensure that the experimental manipulation was effective, post-randomization exclusions based on manipulation performance can introduce selection bias and compromise the equivalence of the randomized groups. Future studies should consider using intention-to-treat analyses alongside per-protocol analyses or employ stronger manipulation designs that minimize the rate of manipulation failure to assess the robustness of the findings. Finally, several general methodological limitations should be noted. Because all measures were self-reported and administered at the same time point immediately after priming, shared method variance may have inflated the correlations among the mediators and the outcome, introducing common-method bias. This also limits the ecological validity of the findings. The reliance on immediate self-report outcomes means that the durability of the observed effects and their translation to actual doctor–patient interactions remain untested. Future research should incorporate behavioral outcome measures, longitudinal follow-ups, and diverse patient samples to strengthen the real-world applicability of these findings.

In terms of practical significance, this study points out that negative meta-stereotypes have a negative impact on the doctor–patient relationship, making people aware of this important factor influencing the relationship. It also reveals the mediating roles of intergroup anxiety and doctor–patient trust, which helps medical institutions optimize services in a targeted manner, improve patients’ experiences, and enhance treatment compliance. The study verifies that imagined intergroup contact can mitigate negative impacts and provides a simple and feasible intervention strategy for improving the doctor–patient relationship. Additionally, it finds that doctors’ emotional care has a greater influence on patients’ attitudes, prompting medical institutions to focus on cultivating doctors’ emotional care capabilities. In the future, it is possible to further explore whether the activation of negative meta-stereotypes has a positive resistance effect on the doctor–patient relationship. It is also necessary to explore from the doctors’ perspective whether the activation of doctors’ negative meta-stereotypes affects the doctor–patient relationship and its influencing mechanism.

## 5. Conclusions

This research investigated the mechanisms through which negative meta-stereotypes impair the doctor–patient relationship and tested an imagined intergroup contact intervention. Across two experiments, negative meta-stereotype activation was associated with heightened intergroup anxiety, diminished doctor–patient trust, and poorer doctor–patient relationship quality. Intergroup anxiety and doctor–patient trust played both independent and chain mediating roles in this relationship, suggesting a sequential emotional-cognitive pathway in which anxiety precedes and contributes to reduced trust. Importantly, only imagined contact with a caring physician—but not purely professional contact—significantly alleviated these negative effects, and this beneficial effect was mediated by doctor–patient trust independently and by intergroup anxiety and doctor–patient trust in a sequential chain. These findings highlight the importance of emotional care in restoring trust and improving doctor–patient relationships, and suggest that targeted, low-cost imagined contact interventions focusing on interpersonal warmth may offer a practical strategy for addressing the relational consequences of negative meta-stereotypes in healthcare settings.

## Figures and Tables

**Table 1:** Descriptive statistics and correlation analysis for Study 1 variables.

Variables	*M* ± *SD*	1	2	3	4	5	6	7
1. Gender	-	1						
2. Education	-	0.088	1					
3. Age	23.050 ± 2.617	−0.251 ***	−0.030	1				
4. Negative meta-stereotypes	4.960 ± 1.118	−0.179 *	−0.185 *	0.114	1			
5. Intergroup anxiety	34.466 ± 9.638	0.117	0.285 **	−0.144 *	−0.335 **	1		
6. Doctor–patient trust	36.152 ± 5.598	−0.112	−0.195 **	0.124	0.507 **	−0.507 **	1	
7. Doctor–patient relationship	60.298 ± 6.321	−0.107	−0.094	0.135	0.497 **	−0.468 **	0.666 **	1

Note. Higher scores on the meta-stereotype measure indicate more positive perceived meta-stereotypes (i.e., the belief that doctors hold more favorable views of patients). Therefore, positive correlations between meta-stereotypes and trust/relationship quality reflect that participants who perceived more positive meta-stereotypes also reported higher trust and better relationship quality. N = 191. * *p* < 0.05, ** *p* < 0.01, *** *p* < 0.001.

**Table 2:** Differences between the two groups of participants on various variables.

Variables	Group	*M* ± *SD*	*F*	*p*	*η_p_* ^2^
Intergroup anxiety	Activation group	36.938 ± 8.031	12.899	<0.001	0.065
	Control group	32.075 ± 10.494			
Doctor–patient trust	Activation group	34.458 ± 5.239	21.892	<0.001	0.104
	Control group	38.021 ± 5.257			
Doctor–patient relationship	Activation group	59.198 ± 6.138	8.078	<0.05	0.041
	Control group	61.670 ± 5.845			

**Table 3:** Standardized regression coefficients for regression models.

Predictors	Model 1 (Dependent Variable: Intergroup Anxiety)			Model 2 (Dependent Variable: Doctor–Patient Trust)			Model 3 (Dependent Variable: Doctor–Patient Relationship)		
	*β*	*SE*	*t*	*β*	*SE*	*t*	*β*	*SE*	*t*
Negative meta-stereotypes	−0.279	0.069	−4.059 ***	0.374	0.062	6.087 ***	0.199	0.059	3.353 **
Intergroup anxiety				−0.371	0.063	−5.894 ***	−0.173	0.060	−2.870 **
Doctor–patient trust							0.475	0.065	7.331 ***
*R* ^2^	0.177			0.386			0.506		
*F*	9.930 ***			23.164 ***			31.265 ***		

Notes. ** *p* < 0.01, *** *p* < 0.001.

**Table 4:** Results of the test of mediated effect.

Effect	Mediation Pathways	Effect Value	95% CI
Direct effect	Negative meta-stereotypes → doctor–patient relationship	0.176	[0.001, 0.351]
Indirect effect 1	Negative meta-stereotypes → intergroup anxiety → doctor–patient relationship	0.048	[0.009, 0.105]
Indirect effect 2	Negative meta-stereotypes → doctor–patient trust → doctor–patient relationship	0.178	[0.104, 0.266]
Indirect effect 3	Negative meta-stereotypes → intergroup anxiety → doctor–patient trust → doctor–patient relationship	0.049	[0.022, 0.082]
Total indirect effects		0.275	
Total effect		0.474	

**Table 5:** Descriptive statistics and correlation analysis for Study 2 variables.

Variables	*M* ± *SD*	1	2	3	4
1. Imagined intergroup contact	-	1			
2. Intergroup anxiety	34.935 ± 10.221	−0.223 *	1		
3. Doctor–patient trust	36.169 ± 5.251	0.246 **	−0.456 **	1	
4. Doctor–patient relationship	60.663 ± 6.985	0.171 **	−0.286 **	0.678 **	1

Note: * *p* < 0.05, ** *p* < 0.01.

**Table 6:** Means, standard deviations, and one-way ANOVA results for intergroup anxiety, doctor–patient trust, and doctor–patient relationship by experimental condition.

Variable	*M* ± *SD*				*F*	*ηp* ^2^
	No-Imagination Group (n = 47)	Imagined Landscape Group (n = 45)	Imagined Professional Group (n = 47)	Imagined Caring Group (n = 45)		
Intergroup anxiety	37.89 ± 10.26	35.8 ± 10.80	34.36 ± 7.98	31.58 ± 10.92	3.199 *	0.051
Doctor–patient trust	34.85 ± 4.56	35.27 ± 6.13	36.21 ± 4.74	38.40 ± 4.91	4.367 **	0.068
Doctor–patient relationship	59.70 ± 7.90	59.89 ± 8.06	59.81 ± 5.58	63.33 ± 5.52	3.003 *	0.048

Note: * *p* < 0.05, ** *p* < 0.01.

**Table 7:** Standardized regression coefficients for the serial mediation model. Notes. *β* = standardized regression coefficient; *SE* = standard error. The model controlled for age, gender, and education level. Coefficients for these covariates are not displayed for brevity but are available upon request. * *p* < 0.05, ** *p* < 0.01, *** *p* < 0.001.

	*β*	*SE*	*t*	*β*	*SE*	*t*	*β*	*SE*	*t*
Imagined intergroup contact	−0.120	0.065	−3.084 **	0.148	0.059	2.524 *	0.012	0.052	0.234
Intergroup anxiety				−0.397	0.066	−6.012 ***	−0.028	0.063	0.447
Doctor–patient trust							0.666	0.066	10.150 ***
*R* ^2^	0.096			0.298			0.465		
*F*	4.742 **			15.113 ***			25.620 ***		

**Table 8:** Relative mediation effects test. *Note:* ^a^ indicates a significant mediating effect.

Mediated Effects Pathways	Estimated Value	95% CI
Using the no-imagination group as a reference		
Imagined landscape → doctor–patient relationship	0.001	[−0.311, 0.313]
Imagined landscape → intergroup anxiety → doctor–patient relationship	−0.005	[−0.058, 0.028]
Imagined landscape → doctor–patient trust → doctor–patient relationship	0.065	[−0.180,0.322]
Imagined landscape → intergroup anxiety → doctor–patient trust → doctor–patient relationship	0.048	[−0.065, 0.173]
Imagined professional → doctor–patient relationship	−0.138	[−0.449, 0.174]
Imagined professional → intergroup anxiety → doctor–patient relationship	−0.010	[−0.069, 0.038]
Imagined professional → doctor–patient trust → doctor–patient relationship	0.124	[−0.110, 0.345]
Imagined professional → intergroup anxiety → doctor–patient trust → doctor–patient relationship	0.085	[−0.019, 0.196]
Imagined caring → doctor–patient relationship	0.093	[−0.232, 0.418]
Imagined Caring → intergroup anxiety → doctor–patient relationship	−0.018	[−0.117, 0.068]
Imagining caring → doctor–patient trust → doctor–patient relationship	0.309 ^a^	[0.080, 0.542]
Imagining caring → intergroup anxiety → doctor–patient trust → doctor–patient relationship	0.162 ^a^	[0.044, 0.295]

## Data Availability

The data presented in this study are available on request from the corresponding author due to privacy and ethical restrictions.
